# Outcomes for patients with EBV-positive PTLD post-allogeneic HCT after failure of rituximab-containing therapy

**DOI:** 10.1038/s41409-023-02127-9

**Published:** 2023-10-21

**Authors:** Gérard Socié, Pere Barba, Arie Barlev, Jaime Sanz, Irene García-Cadenas, Patrice Chevallier, Franca Fagioli, Norma Guzman-Becerra, Deepali Kumar, Per Ljungman, Arnaud Pigneux, Natalia Sadetsky, Lucrecia Yáñez San Segundo, Mazyar Shadman, Jan Storek, Dhanalakshmi Thirumalai, Baodong Xing, Mohamad Mohty

**Affiliations:** 1 Hôpital Saint-Louis, Paris, France; 2 Hospital Universitari Vall Hebron, Universitat Autònoma de Barcelona, Barcelona, Spain; 3 Atara Biotherapeutics, Thousand Oaks, CA USA; 4 Hospital Universitari I Politècnic La Fe, Valencia, Spain; 5 Hematology Division, Hospital de la Santa Creu I Sant Pau, Barcelona, Spain; 6 CHU Hotel Dieu, Nantes, France; 7 Regina Margherita Children’s Hospital, AOU Città della Salute e della Scienza, Turin, Italy; 8 University of Turin, Turin, Italy; 9 Transplant Infectious Diseases and Multi-Organ Transplant Program, University Health Network, Toronto, ON Canada; 10 Karolinska Institutet Huddinge, Stockholm, Sweden; 11 Karolinska Comprehensive Cancer Center, Karolinska University Hospital, Stockholm, Sweden; 12 CHU Bordeaux, Service d’Hématologie Clinique et de Thérapie Cellulaire, Bordeaux, France; 13 Hospital Universitario Marqués de Valdecilla-IDIVAL, Santander, Spain; 14 University of Washington, Seattle, WA USA; 15 Fred Hutchinson Cancer Center, Seattle, WA USA; 16 University of Calgary, Calgary, AB Canada; 17 Sorbonne University, Saint-Antoine Hospital, Paris, France

**Keywords:** Haematological cancer, Haematological cancer

## Abstract

Epstein–Barr virus-positive (EBV^+^) post-transplant lymphoproliferative disease (PTLD) is an ultra-rare and aggressive condition that may occur following allogeneic hematopoietic cell transplant (HCT) due to immunosuppression. Approximately half of EBV^+^ PTLD cases are relapsed or refractory (R/R) to initial rituximab-containing therapy. There are limited treatment options and no standard of care for patients with R/R EBV^+^ PTLD, and little is known about their treatment history and outcomes. We performed a multinational, multicenter, retrospective chart review of patients with R/R EBV^+^ PTLD following HCT to describe patients’ demographic and disease characteristics, treatment history, and overall survival (OS) from rituximab failure. Among 81 patients who received initial treatment with rituximab as monotherapy (84.0%) or in combination with chemotherapy (16.0%), median time from HCT to PTLD diagnosis was 3.0 months and median OS was 0.7 months. Thirty-six patients received a subsequent line of treatment. The most frequent causes of death were PTLD (56.8%), graft-versus-host disease (13.5%) and treatment-related mortality (10.8%). In multivariate analysis, early PTLD onset and lack of response to initial treatment were associated with mortality. This real-world study demonstrates that the prognosis of patients with R/R EBV^+^ PTLD following HCT remains poor, highlighting the urgent unmet medical need in this population.

## Introduction

Post-transplant lymphoproliferative disease (PTLD) is an aggressive and potentially fatal hematologic malignancy that can occur following transplantation due to immunosuppression. Nearly all PTLD cases following hematopoietic stem cell transplant (HCT) are Epstein–Barr virus (EBV)-positive (EBV^+^) and occur as a result of EBV activation in EBV-negative patients who receive a transplant from EBV^+^ donors or due to EBV reactivation in previously infected patients following transplantation [1, 2].

EBV^+^ PTLD is an ultra-rare disease, with an incidence of 1.1–1.7% within the first year after allogeneic HCT [3, 4]. In the USA, there were about 8200 HCTs in 2021, thus resulting in fewer than 150 new PTLD cases per year, and in Europe there were 19,806 HCTs, thus resulting in approximately 275 new cases [5, 6]. The median time to PTLD from HCT is about 2–4 months, with the majority of cases occurring within the first year following transplant, corresponding to recovery of the immune system [3, 7, 8]. The most frequently and consistently identified risk factors for developing EBV^+^ PTLD are prior HCT, post-transplant EBV DNAemia, T-cell depletion ex vivo or in vivo, histocompatibility or EBV serology mismatch between the donor and the recipient, and the use of cord blood [8–16].

Clinical practice treatment guidelines recommend rituximab with or without reduction in immunosuppression (RIS) as pre-emptive therapy for EBV reactivation (based on EBV viral load) and for treatment of EBV^+^ PTLD following HCT [17]. Patients who fail rituximab have poor outcomes with limited treatment options. Although results vary according to protocol, up to 50% of patients with EBV^+^ PTLD post-HCT may experience failure to rituximab-containing treatment [3, 16]. Factors associated with a poor response to rituximab include acute graft-versus-host disease (GvHD) with immunosuppressive drugs, extranodal involvement, the inability to tolerate RIS, and the use of bone marrow graft [7, 18]. The 3-year overall survival (OS) for allogeneic HCT recipients with EBV^+^ PTLD treated with rituximab-containing therapies ranges from 20% to 48% [8, 19, 20], and patients with multiple risk factors experience the worst OS rates [7].

Alternative treatment options for patients with EBV^+^ PTLD post-HCT after failure of initial therapy represent a significant unmet clinical need. Guidelines for subsequent treatment options in patients with relapsed or refractory (R/R) EBV^+^ PTLD post-HCT are based on a limited body of evidence [17], and outcomes following rituximab ± chemotherapy failure are usually poor, with a reported median OS of 33 days [18]. Further, chemotherapy is usually ineffective, with a high treatment-related mortality rate in patients with R/R EBV^+^ PTLD post-HCT [7, 18], which limits treatment options following failure of rituximab. Little information is available regarding the clinical characteristics, treatment patterns, and survival of patients with R/R EBV^+^ PTLD following HCT in a real-world setting. Collation of such data may help inform future treatment decisions and guide how physicians manage these patients in the absence of well-defined, global treatment guidelines.

To address the knowledge gap, we conducted a retrospective chart review at multiple stem cell transplant centers to describe the clinical characteristics and survival of HCT recipients with R/R EBV^+^ PTLD following rituximab ± chemotherapy failure.

## Methods

### Study design and conduct

A multicenter, non-interventional, retrospective chart review of allogeneic HCT recipients with R/R EBV^+^ PTLD following rituximab ± chemotherapy failure was performed. The study was approved by an independent ethics committee, research ethics board, or institutional review board at each center and complied with the Declaration of Helsinki, the International Council for Harmonisation Tripartite Guideline for Good Clinical Practice, and local laws.

### Selection of the study population

A total of 22 sites in Europe (Austria, Belgium, Germany, France, Italy, Spain, and Sweden) and North America (Canada and the USA) contributed data to the study.

Inclusion and exclusion criteria were aligned with the multicenter, open-label, phase III ALLELE trial assessing tabelecleucel in patients with R/R EBV^+^ PTLD following rituximab ± chemotherapy [21]. Eligible patients were HCT recipients who were diagnosed with R/R EBV^+^ PTLD following rituximab ± chemotherapy failure, of any age, and with data records available. PTLD was locally assessed using confirmatory histology or high EBV viremia with clinical and/or radiologic assessment via computed tomography or positron emission tomography. Patients were excluded if they had received cytotoxic T-lymphocytes (CTL), donor lymphocyte infusion (DLI), or had specific PTLD histology of Burkitt, Hodgkin, or T-cell lymphoma.

Existing chart data on patients diagnosed with EBV^+^ PTLD following HCT who received rituximab or rituximab plus chemotherapy between January 2000 and December 2018 and in whom disease was refractory (failed to achieve complete response or partial response) or had relapsed at any point after such therapy were collected. A comprehensive data collection form was developed to capture the heterogeneity of the disease, and electronic case report forms (eCRFs) were developed and utilized through a secured website for study site personnel to submit information. The conduct of the study was standardized, and rigorous procedures to ensure accuracy were followed throughout the data collection process. Data management procedures were implemented following good clinical practice guidelines and included validation and skip patterns to minimize data entry errors, development of guidelines for completion of the eCRFs, and extensive training of study site personnel.

The collected data were entered into a validated database. The data were reviewed manually by trained personnel to ensure data quality, and any data issues identified were addressed through queries and communicated to the sites for resolution. An extensive effort was undertaken to ensure data quality with multiple rounds of medical review to reach resolution. Data management procedures were implemented following Good Clinical Practice guidelines.

### Patient characteristics and outcomes

Demographic information, HCT characteristics, PTLD characteristics, treatment history, and OS data were evaluated. Demographic information included patients’ age (years) and sex (male/female). HCT characteristics included age at HCT, initial diagnosis leading to HCT, the type of allograft used, the stem cell source, and the conditioning regimen used. PTLD characteristics included the time from transplant to PTLD, pre-emptive use of rituximab for PTLD, PTLD histology type, PTLD stage, extranodal sites of PTLD, CD20 marker, Eastern Cooperative Oncology Group (ECOG)/Karnofsky/Lansky score, and the incidence of secondary central nervous system involvement. OS was defined as the time from the index date to the date of death from any cause. OS was assessed using the date of failure to rituximab-containing therapy as the index date, unless otherwise stated. Patients who were lost to follow-up or still alive were censored at the last reported contact or recorded visit date. Cause of death was reported as recorded by the physicians in the case report form.

### Statistical analyses

All continuous variables were summarized using descriptive statistics and all categorical variables were summarized using frequencies and percentages. OS was summarized using the Kaplan–Meier method. Association between several important clinical and demographic variables and mortality was evaluated using Cox proportional hazards multivariate regression analysis. These variables included age (years) at initial PTLD diagnosis, sex, time from transplantation to PTLD diagnosis (days), baseline lactate dehydrogenase (LDH), stage at initial PTLD diagnosis, ECOG performance status, PTLD histology at initial diagnosis, extranodal PTLD sites, pre-emptive use of rituximab for EBV viremia, and response to initial rituximab-containing treatment.

## Results

### Patient demographics and disease characteristics

Medical chart data from 81 patients with R/R EBV^+^ PTLD following rituximab ± chemotherapy failure were analyzed.

Of the 81 included patients, 37 (45.7%) underwent HCT between 2000 and 2010; 44 (54.3%) patients underwent HCT after 2010 (Table 1). The median (minimum–maximum) age at HCT was 48.7 (2–75) years. The most common primary disease leading to HCT was acute myeloid leukemia (32.1%), followed by acute lymphoblastic leukemia (16.0%) and myelodysplastic syndromes (8.6%). Conditioning regimens used prior to HCT included myeloablative conditioning (59.3%) and reduced intensity conditioning (37.0%). Patients received transplants from human leukocyte antigen (HLA)-matched unrelated donors (40.7%), mismatched unrelated donors (33.3%), or matched related donors (12.3%). Stem cells were obtained from peripheral blood mononuclear cells (53.1%), cord blood (25.9%), or bone marrow (11.1%). At the time of HCT, 53 (65.4%) patients were in remission from their primary disease and 26 (32.1%) patients had relapsed disease (data not shown). A total of 17 (21.0%) patients received anti-thymocyte globulin.

**Table 1:** HCT characteristics.

Characteristics	R/R to rituximab ± chemotherapy (*N* = 81)
Sex, *n* (%)	
Male	49 (60.5)
Female	32 (39.5)
Year of HCT, *n* (%)	
2000–2010	37 (45.7)
2010–2018	44 (54.3)
Age at HCT, years	
Median (minimum–maximum)	48.7 (2–75)
Initial diagnosis leading to HCT, *n* (%)	
Acute myeloid leukemia	26 (32.1)
Myelodysplastic syndromes	7 (8.6)
Acute lymphocytic leukemia	13 (16.0)
Non-Hodgkin lymphoma	4 (4.9)
Aplastic anemia	5 (6.2)
Chronic lymphocytic leukemia	4 (4.9)
Chronic myeloid leukemia	4 (4.9)
Multiple myeloma	1 (1.2)
Other	16 (19.8)
Missing	1 (1.2)
Type of allograft, *n* (%)	
Matched related donor	10 (12.3)
Matched unrelated donor	33 (40.7)
Haploidentical	5 (6.2)
Mismatched related donor	3 (3.7)
Mismatched unrelated donor	27 (33.3)
Unknown	2 (2.5)
Stem cell source, *n* (%)	
PBMCs	43 (53.1)
Cord blood	21 (25.9)
Bone marrow	9 (11.1)
Unknown	7 (8.6)
Conditioning regimen used, *n* (%)	
Reduced intensity conditioning	30 (37.0)
Myeloablative conditioning	48 (59.3)
Unknown	2 (2.5)
Anti-T-cell antibody treatment received, *n* (%)	
Yes	17 (21.0)
No	64 (79.0)
Type of anti-T-cell antibody treatment received, *n* (%)	
Anti-thymocyte globulin	17 (100.0)

*HCT* Hematopoietic stem cell transplant, *PBMC* Peripheral blood mononuclear cell, *R/R* Relapsed or refractory.

Patient PTLD disease characteristics are described in Table 2. EBV^+^ viremia was detected in the majority (95.1%) of patients at a median time from HCT of 1.9 months. Seventeen (22.0%) patients were treated pre-emptively with rituximab to prevent PTLD. The median time from HCT to initial PTLD diagnosis was 3.0 months and median age at initial PTLD diagnosis was 49.0 years. Most (74.1%) patients had a baseline LDH of ≥250 U/L. PTLD was diagnosed at an advanced stage (III or IV) in 63 (77.8%) patients. The most common histologic subtype was monomorphic PTLD, which was observed in 52 (64.2%) patients; 18 (22.2%) patients presented with polymorphic PTLD. The most common sites of PTLD involvement were the lymph nodes (62 patients [76.5%]), liver (29 patients [35.8%]), spleen (23 patients [28.4%]), lung (17 patients [21.0%]), and gastrointestinal tract (14 patients [17.3%]) (data not shown). Overall, PTLD involved extranodal sites in 69.1% of patients. CD20 positivity was observed in 52 of 67 patients with available data.

**Table 2:** PTLD disease characteristics.

Characteristics	R/R to rituximab ± chemotherapy (*N* = 81)
Time from HCT to EBV viremia, months	
Median (minimum–maximum)	1.9 (0.0–102.6)
Pre-emptive use of rituximab for PTLD, *n* (%)	
Yes	17 (22.1)
No	60 (77.9)
Age at initial PTLD diagnosis, years	
Median (minimum–maximum)	49.0 (2–75)
Baseline ECOG performance score (only for subjects ≥ 16 years old),^a^ *n* (%)	
<2	8 (22.9)
≥2	27 (77.1)
Baseline elevated LDH,^b^ *n* (%)	60 (74.1)
Time from HCT to PTLD, months	
Median (minimum–maximum)	3.0 (0.8–100.8)
PTLD histology type, *n* (%)	
Early lesions	2 (2.5)
Polymorphic	18 (22.2)
Monomorphic	52 (64.2)
DLBCL	46 (56.8)
Unknown	9 (11.1)
PTLD stage, *n* (%)	
Stage I/II	8 (9.8)
Stage III	17 (21.0)
Stage IV	46 (56.8)
Unknown	10 (12.3)
Extranodal sites of PTLD, *n* (%)	
Yes	56 (69.1)
No	24 (29.6)
Unknown	1 (1.2)
CD20 marker at diagnosis, *n* (%)	
Positive	52 (64.2)
Negative	15 (18.5)
Unknown	14 (17.3)
Secondary CNS involvement, *n* (%)	7 (8.6)

*CNS* Central nervous system, *DLBCL* Diffuse large B-cell lymphoma, *EBV* Epstein–Barr virus, *ECOG* Eastern Cooperative Oncology Group, *HCT* Hematopoietic stem cell transplant, *LDH* Lactate dehydrogenase, *PTLD* Post-transplant lymphoproliferative disease, *R/R* Relapsed or refractory.

^a^Data reported in 35 patients.

^b^LDH levels ≥250 U/L were considered elevated.

### Treatments for PTLD

The median time (minimum–maximum) from PTLD diagnosis to initial treatment was 0.1 (0.0–3.1) months. After diagnosis of PTLD, RIS was reported for 54 (66.7%) patients. Sixty-eight (84%) patients received rituximab alone and 13 (16.0%) received rituximab combined with chemotherapy as their initial treatment for PTLD. Of the 68 patients who received rituximab alone, the median (minimum–maximum) number of doses was 2 (1–9). Thirty-six (44.4%) patients received next-line therapy, with a chemotherapy-containing regimen being most common (32/36). Only four (11.1%) patients who received next-line therapy achieved a durable response of >6 months from the treatment end date; two of these patients subsequently relapsed.

### Overall survival

At the time of chart review, 74 (91.4%) patients had died (Table 3). The most common cause of death was PTLD (56.8%), followed by GvHD (13.5%) and treatment-related mortality (10.8%).

**Table 3:** Treatment-related mortality.

	R/R to rituximab ± chemotherapy (*N* = 81) *n* (%)
Total deaths	74 (91.4)
Cause of death	
PTLD	42 (56.8)
GvHD	10 (13.5)
Treatment-related mortality	8 (10.8)
Sepsis infection	5 (6.8)
Relapsed primary disease leading to HCT	3 (4.1)
Organ rejection/failure	3 (4.1)
Unknown	2 (2.7)
Graft failure	1 (1.4)

*GvHD* Graft-versus-host disease, *HCT* Hematopoietic stem cell transplant, *PTLD* Post-transplant lymphoproliferative disease, *R/R* Relapsed or refractory.

**Fig. 1:**
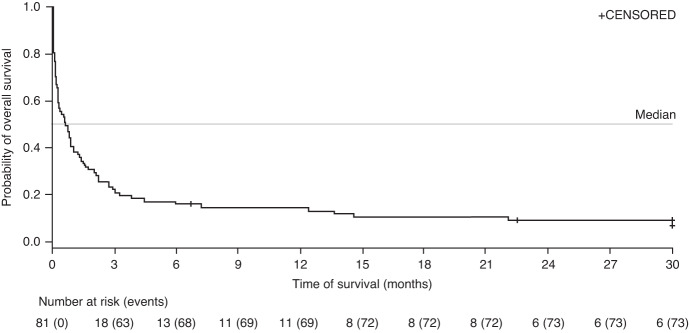
Kaplan–Meier plot of OS from date of R/R to rituximab ± chemotherapy. OS is from the R/R date to the end of follow-up. *OS* Overall survival, *R/R* Relapsed or refractory.

From the date of R/R to rituximab-containing therapy, median (range) follow-up was 0.7 (0.03–107.1) months with a median OS (95% confidence interval [CI]) of 0.7 (0.3–1.0) months. OS (95% CI) at 12 months was 14.7% (8.0–23.3) (Table 4, Fig. 1). In patients who received next-line therapy, median (minimum–maximum) follow-up was 2.0 (0.1–107.1) months with a median OS (95% CI) of 2.0 (1.1–5.5) months from the start date of the next line.

**Table 4:** Overall survival.

	R/R to rituximab ± chemotherapy (*N* = 81)
Median follow-up, months (minimum–maximum)	0.7 (0.03–107.1)
Median OS,^a^ months (95% CI)	0.7 (0.3–1.0)
OS rate,^a^ % (95% CI)	
3 months	22.2 (13.9–31.8)
6 months	16.0 (9.1–24.8)
12 months	14.7 (8.0–23.3)
24 months	9.4 (4.2–17.0)

*CI* Confidence interval, *OS* Overall survival, *R/R* Relapsed/refractory.

^a^From the time of rituximab ± chemotherapy failure leading to R/R Epstein–Barr virus-positive post-transplant lymphoproliferative disease following hematopoietic stem cell transplant.

### Risk factors associated with mortality

A multivariate analysis using the Cox proportional hazards ratio regression model was conducted to determine if key baseline characteristics were associated with mortality (Table 5). Early PTLD onset (defined as ≤100 days after HCT; hazard ratio (HR) [95% CI]: 2.33 [1.25–4.37]) and a best overall response of stable or progressive disease (i.e., non-responders) following initial therapy (HR [95% CI]: 3.74 [1.81–7.70]) were significantly associated with mortality.

**Table 5:** Multivariate analysis of potential factors associated with mortality.

	R/R to rituximab ± chemotherapy, *N*	HR (95% CI)	*p-*value
Age at initial PTLD diagnosis			
<60 years (low risk)	69	ref	
≥60 years (high risk)	12	1.22 (0.59–2.51)	0.5943
Sex			
Male	49	ref	
Female	32	1.10 (0.61–1.99)	0.7566
Elevated baseline LDH (≥250 U/L)			
No	11	ref	
Yes	60	2.51 (0.93–6.82)	0.0706
Missing	10	2.56 (0.75–8.76)	0.1329
Region			
North America	24	ref	
Europe	57	0.99 (0.45–2.21)	0.9852
PTLD stage at initial diagnosis			
Stage 1 or 2	8	ref	
Stage 3 or 4	63	0.86 (0.34–2.19)	0.7563
Missing	10	0.69 (0.21–2.26)	0.5414
PTLD histology at initial diagnosis			
All other types	29	ref	
Monomorphic	52	0.72 (0.42–1.23)	0.2322
Time from HCT procedure to initial PTLD diagnosis	81	0.99 (0.96–1.02)	0.5952
PTLD onset^a^			
Late	37	ref	
Early	44	2.33 (1.25–4.37)	0.0081
Extranodal sites of PTLD			
No or unknown	25	ref	
Yes	56	1.00 (0.52–1.92)	0.9986
Pre-emptive use of rituximab for PTLD			
No or unknown	64	ref	
Yes	17	0.85 (0.41–1.75)	0.6551
Response to initial therapy^b^			
Responders	15	ref	
Non-responders	66	3.74 (1.81–7.70)	0.0004
Total number of systemic treatments			
1	43	ref	
2	29	0.41 (0.07–2.55)	0.3409
3	9	0.36 (0.05–2.75)	0.3237
Received next line of therapy			
No	45	ref	
Yes	36	0.53 (0.09–3.18)	0.4832
ECOG/Karnofsky/Lansky score			
<2/≥70/≥70 (low risk)	13		
≥2/<70/<70 (high risk)	34	1.57 (0.70–3.51)	0.2755
Missing	34	0.72 (0.31–1.70)	0.4519

*CI* Confidence interval, *ECOG* Eastern Cooperative Oncology Group, *HCT* Hematopoietic stem cell transplant, *HR* Hazard ratio, *LDH* Lactate dehydrogenase, *PTLD* Post-transplant lymphoproliferative disease, *ref* Reference, *R/R* Relapsed or refractory.

^a^Early PTLD onset is defined as ≤100 days after HCT, whereas late PTLD onset is defined as >100 days after HCT.

^b^Responders were patients who achieved a complete or partial response to initial therapy. Non-responders were patients who had stable disease or progressive disease following initial therapy.

## Discussion

Medical literature describing clinical outcomes in patients with R/R EBV^+^ PTLD is limited; available data reporting the experience of a few patients who receive a subsequent treatment after rituximab indicate very poor outcomes [22, 23]. This retrospective chart review is the first to describe the survival of HCT recipients with EBV^+^ PTLD following rituximab ± chemotherapy failure. We observed that patients with R/R EBV^+^ PTLD post-HCT experience poor survival, with a median OS of 0.7 months from the time of initial treatment failure, and only 14.7% of patients surviving at 12 months, with the majority dying because of PTLD-related mortality (56.8%) and treatment-related mortality (10.8%), while patients who received next-line therapy had a median OS of 2.0 months from the initiation of the next line, thus demonstrating an urgent unmet medical need in this patient population. These data can be used as a benchmark for future interventional studies in this disease setting.

Given such poor outcomes, we sought to identify factors associated with mortality in patients with R/R EBV^+^ PTLD. Identification of such factors may help delineate high-risk patients and ultimately improve early detection and treatment options for patients with R/R EBV^+^ PTLD. Our multivariate analysis evaluated whether age at initial PTLD diagnosis, sex, region, baseline LDH, stage at initial PTLD diagnosis, PTLD histology at initial diagnosis, time from HCT procedure to initial PTLD diagnosis, extranodal PTLD sites, pre-emptive use of rituximab for EBV viremia, response to initial rituximab-containing treatment, number of systemic treatments, receipt of next-line therapy and ECOG/Karnofsky/Lansky score were associated with survival in patients with R/R EBV^+^ PTLD. In this multivariate analysis, two factors were significantly associated with mortality: early PTLD onset (≤100 days after HCT) and the lack of response to initial therapy. To our knowledge, this is the first time early PTLD onset and a lack of response to initial therapy has been associated with an elevated risk of mortality. There was a suggestion of an association between elevated baseline LDH (≥250 U/L) and mortality also observed, which is unsurprising as previous analyses in patients with PTLD following HCT or solid organ transplant have reported that elevated LDH was associated with a lack of response to initial treatment and reduced OS [4].

Our study is the first to assess patients with R/R EBV^+^ PTLD in the HCT setting, albeit using a retrospective study design. Limitations associated with retrospective observational studies are that they may be difficult to establish causality and they may also be subject to certain biases. However, in the setting of a rare disease requiring urgent care, a prospective cohort design is likely to be impractical. We focused on OS as the outcome of interest, given that it can be assessed accurately in a real-world setting, whereas other outcomes such as response rate have limitations in real-world settings, such as the lack of standardized modalities for evaluating response to treatment, temporal changes in treatment and technology, variable evaluation frequencies, and variability in physician practice. Patients who received DLI or EBV- or multivirus-specific CTL therapy after PTLD diagnosis (an option available for several years) or those with a history of Burkitt, Hodgkin, or T-cell lymphoma were excluded in our study protocol in order to align with the phase III ALLELE trial; thus, our results are only representative of patients with PTLD for whom such therapy is not available. Given these considerations, our study provides significant insights for this high unmet need population.

A key strength of this study is that it is the largest and most comprehensive multinational chart review of patients with R/R EBV^+^ PTLD following failure of rituximab-containing therapy. A further strength is that careful thought was given to the identification of important prognostic factors and to minimization of missing data. Our study evaluated charts recorded between 2000 and 2018, during which time no novel therapies were approved that may have impacted the study findings. Data were not available after this time period.

Our study confirmed the lack of adequate treatment options that target the underlying pathology of PTLD. As there were no therapies approved for the management of PTLD from 2000 to 2018, rituximab-containing therapy became an established treatment option, although not all patients respond. Survival outcomes are worse for patients without a response to rituximab. HCT patients may also be frail and require a subsequent therapy that has a tolerable safety profile after failure of rituximab. Lack of a standard of care may contribute to differences in treatment patterns, which further limits comparability and generalizability between small studies and hinders research advances urgently needed by this subset of patients. The outcomes associated with the use of rituximab ± chemotherapy for patients with R/R EBV^+^ PTLD post-HCT described in this retrospective chart review underline the unmet need for new treatment options that are safe and effective in this patient group.

In summary, this retrospective chart review has demonstrated that patients with R/R EBV^+^ PTLD have limited therapy options, resulting in poor outcomes. Our analysis confirms the high unmet medical need in such patients post-HCT in whom EBV^+^ PTLD relapses or becomes refractory to initial rituximab-containing treatment.

## Data Availability

Aggregate data analyses generated during this study are included in this published article. Patient-level data are owned by individual sites, but due to the rare nature of EBV^+^ PTLD, will not be shared to ensure patient confidentiality.

## References

[1] Dierickx D, Habermann TM (2018). Post-transplantation lymphoproliferative disorders in adults. N Engl J Med.

[2] Ibrahim HA, Naresh KN (2012). Posttransplant lymphoproliferative disorders. Adv Hematol.

[3] Garcia-Cadenas I, Yanez L, Jarque I, Martino R, Perez-Simon JA, Valcarcel D (2019). Frequency, characteristics, and outcome of PTLD after allo-SCT: a multicenter study from the Spanish group of blood and marrow transplantation (GETH). Eur J Haematol.

[4] Dierickx D, Tousseyn T, Sagaert X, Fieuws S, Wlodarska I, Morscio J (2013). Single-center analysis of biopsy-confirmed posttransplant lymphoproliferative disorder: incidence, clinicopathological characteristics and prognostic factors. Leuk Lymphoma.

[5] Passweg JR, Baldomero H, Ciceri F, Corbacioglu S, de la Cámara R, Dolstra H, et al. Hematopoietic cell transplantation and cellular therapies in Europe 2021. The second year of the SARS-CoV-2 pandemic. A report from the EBMT Activity Survey. Bone Marrow Transplant. 2023;58:647–58. 10.1038/s41409-023-01943-3 PMC9987384 36879108

[6] Bolon YT, Atshan R, Allbee-Johnson M, Estrada-Merly N, Lee SJ. Current use and outcome of hematopoietic stem cell transplantation: CIBMTR US summary slides. 2022. https://cibmtr.org/CIBMTR/Resources/Summary-Slides-Reports. Accessed 23 June 2023.

[7] Styczynski J, Gil L, Tridello G, Ljungman P, Donnelly JP, van der Velden W (2013). Response to rituximab-based therapy and risk factor analysis in Epstein Barr Virus-related lymphoproliferative disorder after hematopoietic stem cell transplant in children and adults: a study from the Infectious Diseases Working Party of the European Group for Blood and Marrow Transplantation. Clin Infect Dis.

[8] Uhlin M, Wikell H, Sundin M, Blennow O, Maeurer M, Ringden O (2014). Risk factors for Epstein-Barr virus-related post-transplant lymphoproliferative disease after allogeneic hematopoietic stem cell transplantation. Haematologica..

[9] Sundin M, Le Blanc K, Ringden O, Barkholt L, Omazic B, Lergin C (2006). The role of HLA mismatch, splenectomy and recipient Epstein-Barr virus seronegativity as risk factors in post-transplant lymphoproliferative disorder following allogeneic hematopoietic stem cell transplantation. Haematologica..

[10] Rasche L, Kapp M, Einsele H, Mielke S (2014). EBV-induced post transplant lymphoproliferative disorders: a persisting challenge in allogeneic hematopoetic SCT. Bone Marrow Transplant.

[11] Landgren O, Gilbert ES, Rizzo JD, Socie G, Banks PM, Sobocinski KA (2009). Risk factors for lymphoproliferative disorders after allogeneic hematopoietic cell transplantation. Blood..

[12] Kalra A, Roessner C, Jupp J, Williamson T, Tellier R, Chaudhry A (2018). Risk factors for post-transplant lymphoproliferative disorder after thymoglobulin-conditioned hematopoietic cell transplantation. Clin Transplant.

[13] Fujimoto A, Hiramoto N, Yamasaki S, Inamoto Y, Uchida N, Maeda T (2019). Risk factors and predictive scoring system for post-transplant lymphoproliferative disorder after hematopoietic stem cell transplantation. Biol Blood Marrow Transplant.

[14] Enok Bonong PR, Zahreddine M, Buteau C, Duval M, Laporte L, Lacroix J (2021). Factors associated with post-transplant active Epstein-Barr virus infection and lymphoproliferative disease in hematopoietic stem cell transplant recipients: a systematic review and meta-analysis. Vaccines.

[15] Curtis RE, Travis LB, Rowlings PA, Socie G, Kingma DW, Banks PM (1999). Risk of lymphoproliferative disorders after bone marrow transplantation: a multi-institutional study. Blood..

[16] Kinzel M, Dowhan M, Kalra A, Williamson TS, Dabas R, Jamani K (2022). Risk factors for the incidence of and the mortality due to post-transplant lymphoproliferative disorder after hematopoietic cell transplantation. Transplant Cell Ther.

[17] Styczynski J, van der Velden W, Fox CP, Engelhard D, de la Camara R, Cordonnier C (2016). Management of Epstein-Barr Virus infections and post-transplant lymphoproliferative disorders in patients after allogeneic hematopoietic stem cell transplantation: Sixth European Conference on Infections in Leukemia (ECIL-6) guidelines. Haematologica..

[18] Fox CP, Burns D, Parker AN, Peggs KS, Harvey CM, Natarajan S (2014). EBV-associated post-transplant lymphoproliferative disorder following in vivo T-cell-depleted allogeneic transplantation: clinical features, viral load correlates and prognostic factors in the rituximab era. Bone Marrow Transplant.

[19] Fujimoto A, Suzuki R (2020). Epstein-Barr virus-associated post-transplant lymphoproliferative disorders after hematopoietic stem cell transplantation: pathogenesis, risk factors and clinical outcomes. Cancers.

[20] Hoegh-Petersen M, Goodyear D, Geddes MN, Liu S, Ugarte-Torres A, Liu Y (2011). High incidence of post transplant lymphoproliferative disorder after antithymocyte globulin-based conditioning and ineffective prediction by day 28 EBV-specific T lymphocyte counts. Bone Marrow Transplant.

[21] ClinicalTrials.gov. NCT03394365. Tabelecleucel for solid organ or allogeneic hematopoietic cell transplant participants with Epstein-Barr virus-associated post-transplant lymphoproliferative disease (EBV+ PTLD) after failure of rituximab or rituximab and chemotherapy. 2017. https://ClinicalTrials.gov/show/NCT03394365. Accessed 8 September 2022.

[22] Xu H, Forsythe A, Barlev A, Rashid N, Watson C (2018). A systematic literature review of real-world evidence in post-transplant lymphoproliferative disorder. Blood..

[23] Hou HA, Yao M, Tang JL, Chen YK, Ko BS, Huang SY (2009). Poor outcome in post transplant lymphoproliferative disorder with pulmonary involvement after allogeneic hematopoietic SCT: 13 years’ experience in a single institute. Bone Marrow Transplant.

